# Anorexia nervosa is linked to reduced brain structure in reward and somatosensory regions: a meta-analysis of VBM studies

**DOI:** 10.1186/1471-244X-13-110

**Published:** 2013-04-09

**Authors:** Olga E Titova, Olof C Hjorth, Helgi B Schiöth, Samantha J Brooks

**Affiliations:** 1 Department of Neuroscience, Uppsala University, Box 593, Husargatan 3, Uppsala, Sweden

**Keywords:** VBM, ALE, Anorexia nervosa, Gray matter, White matter, Cerebrospinal fluid

## Abstract

**Background:**

Structural imaging studies demonstrate brain tissue abnormalities in eating disorders, yet a quantitative analysis has not been done.

**Methods:**

In global and regional meta-analyses of 9 voxel-based morphometry (VBM) studies, with a total of 228 eating disorder participants (currently ill with anorexia nervosa), and 240 age-matched healthy controls, we compare brain volumes using global and regional analyses.

**Results:**

Anorexia nervosa (AN) patients have global reductions in gray (effect size = −0.66) and white matter (effect size = −0.74) and increased cerebrospinal fluid (effect size = 0.98) and have regional decreases in left hypothalamus, left inferior parietal lobe, right lentiform nucleus and right caudate, and no significant increases. No significant difference in hemispheric lateralization was found.

**Conclusions:**

Global and regional meta-analyses suggest that excessive restrained eating as found in those with anorexia nervosa coincides with structural brain changes analogous to clinical symptoms.

## Background

Abnormal eating behaviours are commonplace in modern societies; from psychiatric restrained eating and yo-yo dieting to epidemic overeating. An abundance of hyper stimulating advertising and easily-obtainable high energy palatable food likely encourages dysfunctional cognitive biases that prompt pathological eating behaviours [1]. Imbalances in energy intake caused by restrained or overeating are known to influence cognitions over many domains [2], and long-term alterations in energy intake are also associated with brain volume changes [3]. For example, acute adolescent onset and prolonged lifetime starvation, as in those with anorexia nervosa (AN) is associated with global brain volume reductions [4,5], which can be reversed following long-term regular normal eating [6]. However, gray matter reduction has also been shown to persist in those recovered from, but with a history of AN [7]. Additional to gray matter changes, reduced white matter and increased cerebrospinal fluid has been observed consistently in many studies of those with AN [2,8-16]. Regional brain differences in AN are less consistent, although gray matter alterations have been shown in the anterior cingulate cortex, hippocampus and amygdala [17-19]. Against this background, a meta-analysis of previous studies examining brain volume alterations in those with abnormal eating behaviour would strengthen our understanding of the neural underpinnings of AN.

Magnetic Resonance Imaging (MRI) is a technique that can be used to measure brain volume differences, often with a voxel-by-voxel statistically automated technique known as voxel-based morphometry (VBM) [20]. The VBM approach has advantages over other methods, such as manual tracing, a time-consuming technique susceptible to human error, because VBM uses standardized anatomical measures of voxel-wise estimations and parametric mapping to determine tissue-type probabilities, and is quicker to conduct statistical estimations between groups on a whole brain or region of interest basis than some other software approaches.

In a previously published qualitative review of VBM studies in eating disorders [21], it was advised that an important task for future studies is to address whether starvation or irregular energy intake results in the loss of brain tissue. In this previous review, the authors focused on a limited number of heterogeneous studies of eating disorders (e.g. anorexia nervosa, recovered anorexia nervosa and bulimia nervosa) and therefore wisely did not attempt to conduct a meta-analysis. However, unlike the previous meta-analysis, in our novel meta-analysis, we focus on a quantitative meta-analysis of global and regional differences between patients with AN and healthy controls. Furthermore, in contrast to the previous review, we do not include studies that measure those who have recovered from AN, because we aim to conduct an analysis of the effects of current low food intake on brain volume. Additionally, in contrast to the previous review, we conduct extensive meta-analyses on global and regional brain volume differences between AN versus healthy controls. Thus, here we conduct for the first time an extensive meta-analyses of global and regional brain volume differences in AN vs. healthy controls.

## Methods

### Definition of participants with anorexia nervosa (AN)

Our main aim was to measure the effects of food intake amounts on brain volume in those who excessively restrain their food intake, e.g. those with a formal clinical diagnosis of anorexia nervosa (AN). To define those who had a diagnosis of AN we included studies of AN patients that had been diagnosed by the Diagnostic and Statistical Manual Version 4 Revised (DSM-IVR) criteria [22]. There were not enough studies to enable distinct analyses between subtypes of AN (e.g. restrained vs binge-purging), however, by virtue of their low weight (a Body Mass Index [BMI] ≤ 17.5) all participants in these studies had severely restricted food intake.

### Searching

#### Inclusion and exclusion criteria

PubMed, Medline, ScienceDirect, Ovid, Web of Science, and Google Scholar were used to search for VBM studies that examined people who had restrained eating patterns, specifically, people currently ill with AN. We used the following keywords for the search: “anorexia” and “anorexia nervosa”, “eating disorders”, “food”, “MRI”, “magnetic resonance imaging”, “restraint”, “restrained eating”, “VBM”, “voxel based morphometry”. The inclusion criteria were: a) MRI-based approach and not other brain imaging modalities (e.g. Positron Emission Tomography, [PET], Single Photon Emission Computed Tomography [SPECT]) in people with AN and healthy (normal weight) controls, b) cross-sectional case–control studies, c) studies published in the last decade between January 2002 and January 2012, d) published in peer-reviewed journals, e) original articles written in English. For regional brain volume meta-analyses we considered only studies that reported neural activation coordinates in Montreal Neurological Institute (MNI) or Talairach space (Talairach and Tournoux, 1988). MNI coordinates were converted into Talairach for meta-analyses using the conversion tool in GingerALE (http://www.brainmap.org/ale), so that the brain measures would be congruent with other brain imaging studies in our laboratory.

We excluded studies (n = 6) because they were not written in English, were case reports with one participant only [23], considered participants who had recovered from an eating disorder, if no data were available on global brain volumes or global brain volumes were given as a fraction score. We excluded studies from Activation Likelihood Estimation (ALE) meta-analysis (see below) if no data were available on the Talaraich (Talairach and Tournoux, 1988) or Montreal Neurological Institute (MNI) activation foci coordinate (and if we were unable to contact the author) or if white matter foci coordinates were presented only. See Additional file 1: Table S1 for a list of excluded studies.

### The activation likelihood estimation (ALE) approach

ALE is a modeling technique used for the estimation of consistent brain changes across different imaging studies [24]. ALE modeling uses the foci coordinates reported in each study to create a 3-dimensional Gaussian kernel to provide a modeled activation (MA) map for each experiment. The position of foci can be a consequence of between-study variances, such as the different templates used, or the differences between participants, and as such these two main issues are considered in the parameters of the kernel. This is done by weighting the foci reported by the number of participants in each study. Finally, the MA maps for each study are combined for each separate meta-analysis, creating an experimental ALE map. This is tested against the null hypothesis that there is random variation in relation to the spatial orientation of neural activation for the specific meta-analysis (e.g. gray matter decrease in restrained eaters), but that the within-study variation is fixed. A random effects model is employed by the ALE analysis technique, which assumes a higher than chance likelihood of consensus between different experiments, but not in relation to activation variance within each study. The null distribution map is permuted by the number of studies that constitute each meta-analysis. To correct for multiple comparisons, we used a threshold of p < 0.05 False Discovery Rate (FDR), and chose a minimum cluster size of 100 mm3 in line with a recently published fMRI ALE meta-analysis [25]. An anatomical image overlay program Mango (Creators, Jack Lancaster, Michael Martinez: http://ric.uthscsa.edu/mango) was used to illustrate the results of ALE meta-analyses.

### Selected studies for meta-analyses

We found 15 studies that were initially considered for inclusion in the meta-analysis. For the *global brain* analysis, 8 of these studies did not meet the eligibility criteria and were excluded. Of these studies, 2 were review articles, 3 reported the change in gray matter volume in recovered anorectic patients who we did not include in this review, in 1 study only fractions of global brain volumes were presented, in 1 study SPM was not used, and 1 study was a case report with one participant only (see Additional file 1: Table S1). For the *regional brain* analysis that is, the ALE, 8 studies were excluded. Of these studies, 2 were review articles, 3 were not included in the ALE meta-analysis because they did not provide details of Talairach or MNI peak activation coordinates, and it was not possible to gain contact with the authors, 2 studies reported change in gray matter volume in patients recovered from anorexia nervosa, 1 study was a case report with one participant only.

Four studies were included in both Global and ALE meta-analysis and 4 publications were considered only for either global or ALE analysis. Thus, there were 9 studies in total. 7 studies contributed to a meta-analysis of global brain volumes, and 7 that were eligible for the ALE meta-analysis (see Table 1 and Table 2). It is of note that some studies contributed to both global analyses and ALE, while others only contributed to either global or ALE. Three global brain volume analyses using forest plots were done to illustrate differences in gray matter, white matter and cerebrospinal fluid volumes in patients with AN compared to healthy controls). A separate regional brain volume analysis using brain maps were conducted, representing decreased regional brain volume differences in patients with AN vs. controls.

**Table 1:** MRI studies included in the global meta-analyses (n = 7)

**Study**			**Sample**			
	**Author:**	**Type of eating disorder**	**Eating disorder**		**Controls**	
			**n**	**Age (years)**	**n**	**Age (years)**
1	Suchan et al., 2010	Anorexia nervosa	15	26.8 ± 8.4	15	29.5 ± 8.2
			15 female		15 female	
2	Gaudio et al., 2011	Anorexia nervosa in adolescents	16	15.2 ± 1.7	16	15.1 ± 1.5
			16 female		16 female	
3	Boghi et al., 2011 (A)	Anorexia nervosa, patients with shorter disease duration	10	21.4 ± 2.5	13	22.85 ± 1.46
			10 female		13 female	
3	Boghi et al., 2011 (B)	Anorexia nervosa, patients with longer disease duration	11	35.9 ± 9.3	14	38.2 ± 5.1
			11 female		14 female	
4	Castro-Fornieles et al., 2009	Adolescent anorexia nervosa	12	14.5 ± 1.5	9	14.6 ± 3.2
			11 female		8 female	
			1 male		1 male	
5	Swayze et al., 2002	Anorexia nervosa (men and women)	18	25.1 ± 7.3	18	26.0 ± 7.6
			12 female		12 female	
			6 male		6 male	
6	Roberto et al., 2011	Anorexia nervosa	32	26.9 ± 6.4	21	25.0 ± 3.2
			32 female		21 female	
7	Friederich et al., 2012	Anorexia nervosa	12	24.3 ± 6.2	14	25.6 ± 3.7
			12 female		14 female	
			**126**		**120**	

**Table 2:** **MRI studies included in *regional meta-analyses *(n = 7)**

**Study**						**Sample**			
	**Author:**	**Type of eating disorder**	**SPM version**	**foci**	**MRI analysis**	**Eating disorder**		**Controls**	
						**n**	**Age (years)**	**n**	**Age**
1	Joos et al., 2010	Anorexia nervosa	SPM8	7	WB	12	25.0 ± 4.8	18	26.9 ± 5.7
						12 female		18 female	
2	Suchan et al., 2010	Anorexia nervosa	SPM5	2	WB	15	26.8 ± 8.4	15	29.5 ± 8.2
						15 female		15 female	
3	Gaudio et al., 2011	Anorexia nervosa in adolescents	SPM2	3	WB	16	15.2 ± 1.7	16	15.1 ± 1.5
						16 female		16 female	
4	Boghi et al., 2011	Anorexia nervosa, patients with shorter and longer disease duration	SPM2	19	WB	21	29 ± 10	27	30.8 ± 8.7
						21 female		27 female	
5	Castro-Fornieles et al., 2009	Adolescent anorexia nervosa	SPM5	3	WB	12	14.5 ± 1.5	9	14.6 ± 3.2
						11 female		8 female	
						1 male		1 male	
6	Brooks S. et al., 2011	Anorexia nervosa	SPM5	5	WB	14	26 ± 1.9	21	26 ± 2.1
						14 female		21 female	
7	Friederich et al., 2012	Anorexia nervosa	SPM5	8	WB	12	24.3 ± 6.2	14	25.6 ± 3.7
						12 female		14 female	
				**47**		**102**		**120**	

### Analyses of brain volume differences

Magnetic resonance imaging (MRI) is a brain imaging technique which can measure function and structure in high resolution. In studies of eating disorders MRI can detect structural brain changes such as brain tissue or atrophy, increase of brain tissue, or simply differences in volume between groups and time points. Moreover, MRI studies can examine whether brain volume changes linked to clinical syndromes are reversible and whether they are associated with the duration of the disease. Voxel Based Morphometry (VBM) is one MRI technique that compares a proxy measurement for gray matter volume, based on gray matter density volumes and the modulation step (normalization to template) on a voxel-by-voxel basis between two groups of subjects. For a more detailed description see [20].

For the purposes of the present review only cross-sectional MRI studies in people with AN and healthy (normal weight) controls were included. MRI studies generally analyze contrasts either using a Whole Brain (WB) or a Region of Interest (ROI) approach. A WB analysis means that reported significant regional structural differences are derived by comparing areas of activation globally across the whole brain, and unlike ROI analysis, is not limited by specific brain regions that have been previously determined (often based on previous literature). Conversely, a ROI analysis means that a mask or small volume correction is used, to either include or exclude a region of the brain during statistical analysis. We included in meta-analysis only foci of MRI studies derived from WB not ROI analysis. In studies where coordinates from both WB and ROI analysis were provided, we included also only foci from WB.

#### Whole brain analysis

Whole brain (WB) analyses were done using MIX 2.0 PRO for Microsoft Excel (BioStatXL) after extracting global brain data for gray matter, white matter and cerebrospinal fluid from each paper that compared people with anorexia nervosa (AN) with healthy controls. The data we extracted was volumetric data that had been corrected by the authors of the paper for global brain size. Forest plots were used to illustrate the results of the global brain volume analyses.

##### Anorexia nervosa (AN)

Seven separate studies reported global brain tissue volume differences between people with AN and healthy controls [4,5,16,26-29]. See Table 1. Boghi et al. (2011) examined people with AN, with a disease duration of less than 3 years and more than 9 years. In WB analysis these subgroups were considered separately due to different age-matched healthy controls groups [4]. Thus, 126 subjects with AN and 120 healthy controls were included in WB analyses.

#### Regional brain analysis

Regional brain volume meta-analyses were done by extracting regional foci data (MNI or Talairach coordinates) from each paper and using ALE [30-32] to create brain maps of surviving significant regional differences across studies.

##### Anorexia nervosa (AN)

Seven VBM studies of patients with AN were included in ALE meta-analysis [4,5,7,26,27,29,33]. In one follow-up study [27] we only included the baseline data. Anorectic patients with disease duration less than 3 years and more than 9 years in the study of Boghi et al. (2011) were included in ALE meta-analysis, a choice buoyed by the reports that differences in brain volume were similar at both time points [4]. Thus, 47 separate foci, 102 subjects with AN and 120 healthy controls were included in regional brain analyses.

We acquired some information on global brain tissue volumes and/or activation foci coordinates and/or T scores by direct contact with the following authors: Gaudio et al. (2011). Intensity of T scores reported in VBM studies was evaluated separately in an attempt to overcome one of the major flaws of ALE analysis (e.g. that it does not currently take into account the intensity of the difference between groups) [30]. To do this, we calculated the average regional T scores in lobar regions, e.g. frontal, temporal, parietal, occipital and midbrain.

#### Frequency data

We also analyzed the number of studies demonstrating regional gray matter decrease and gray matter increase in the following brain regions: frontal, temporal, parietal, and occipital lobes as well as in mid-brain for people with AN. We also examined whether there was any study bias with regard to lateralization of brain structure differences, by counting the number of studies reporting left or right hemisphere structural differences in the above brain regions.

### Statistical analysis

Meta-analysis of global brain volumes was performed using MIX 2.0. Pro software for Excel, version 2.0.1.4. (Biostat XL, 2011). Regional analysis of structural brain differences was done using ALE.

## Results

### General overview of studies

For study information in the global and regional analyses, see Tables 1 and 2 respectively. For demographic information on study participants for both global and regional (ALE) brain volume analysis, see Table 3.

**Table 3:** Summary of demographic information across studies

	**Patients with anorexia nervosa**		**Healthy control subjects**	
	*Global analysis*	*ALE analysis*	*Global analysis*	*ALE analysis*
Number of males/females (n)	7/119	1/101	7/113	1/119
Age (mean, S.D.)	23.12 (5.84)	22.97 (5.75)	23.80 (6.47)	24.07 (6.57)
BMI (mean, S.D.)	15.12 (1.02)	15.42 (0.68)	21.70 (1.40)	21.30 (0.65)

**Figure 1:**
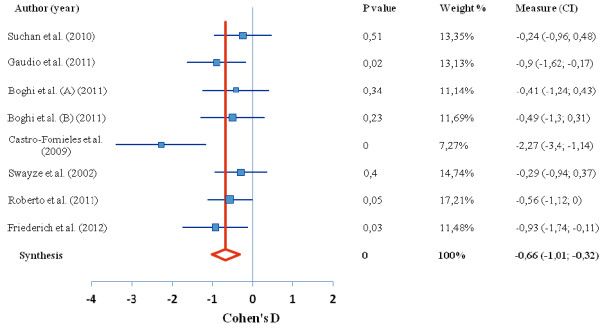
**Difference in GM volume between anorexia nervosa and control group.** Forest plot depicting the difference in global grey matter volume between patients with AN and healthy controls. Authors of study are given on left, red line indicates median, blue boxes indicate separate studies; p value = statistical significance threshold; weight % as adjusted for study population size; CI = confidence intervals.

**Figure 2:**
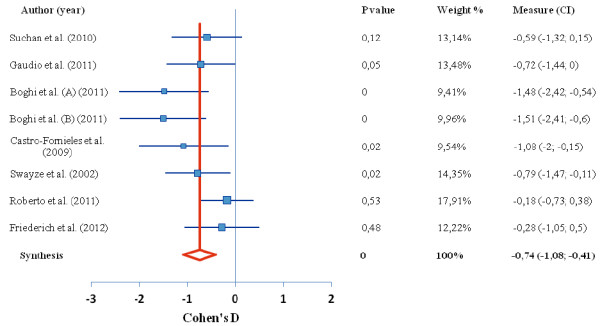
**Difference in WM volume between anorexia nervosa and control group.** Forest plot depicting the difference in global white matter volume between patients with AN and healthy controls. Authors of study are given on left, red line indicates median, blue boxes indicate separate studies; p value = statistical significance threshold; weight % as adjusted for study population size; CI = confidence intervals.

**Figure 3:**
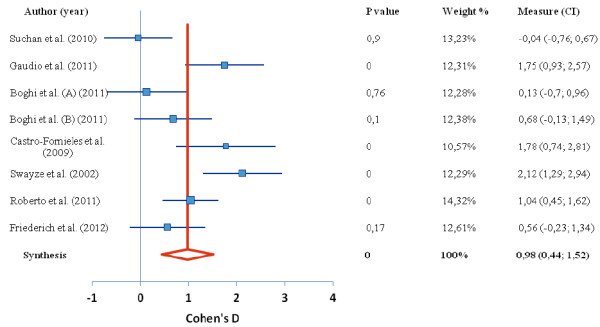
**Difference in CSF volume between anorexia nervosa and control group.** Forest plot depicting the difference in global cerebrospinal fluid volume between patients with AN and healthy controls. Authors of study are given on left, red line indicates median, blue boxes indicate separate studies; p value = statistical significance threshold; weight % as adjusted for study population size; CI = confidence intervals.

Of the 15 studies found in our search, 9 studies contributed to 4 meta-analyses reported here. In the first meta-analysis of the difference in global brain tissue volumes in patients with AN, 7 studies were included, with a total 126 patients and 120 healthy controls. In the second ALE meta-analysis, representing the regional gray matter volume decrease in those with AN (there was only 1 study reporting gray matter increase), 7 studies were included, with a total 102 patients and 120 controls; In the third analysis, we examined average T score intensity and 5 studies were included. Tables 1 and 2 show the complete overview of the included studies. The global brain tissue volumes analysis is showed in Figures 1, 2, 3. The clusters that were significant at the False Discovery Rate (FDR) and 100 mm3 voxel thresholds are reported in Table 4. The following sections are separated into the 4 meta-analyses conducted as described above.

**Table 4:** Locations of centred Talairach peak coordinates with significant ALE values for the MRI studies included in the meta-analysis

**Cluster**^**a**^	**Anatomical label**^**b**^	**Peak voxel coordinates**^**c**^			**Cluster size (mm**^**3**^**)**	**ALE value(x10**^**-2**^**)**	**No. of foci**
		*x*	*y*	*z*			
*Patients with anorexia nervosa* vs. *healthy controls. Gray matter decrease (n =7)*							
1	Hypothalamus	−2	−3	−10	560	1.53	3
2	L Inferior Parietal lobe. Brodmann area 39	−49	−51	9	200	1.08	2
3	R Lentiform nucleus	1 2 2	7	6	128	1.02	2
4	R Caudate	29	1	−8	120	0.97	2

^a^ ALE clusters at p < 0.05 (FDR threshold correction for multiple comparisons, cluster size > 100 mm^3^).

^b^ L, left hemisphere, R, right hemisphere.

^c^ Voxel coordinates are inTalairach space.

#### Meta-analysis one: Global brain analyses in females with AN

Global brain analyses between AN patients versus healthy controls revealed a significant decrease in global gray matter volume, with a medium effect size of −0.66 representing a very large decrease. White matter volume revealed a medium effect size of −0.74 representing a very large decrease. Finally, the global analysis revealed an increase in cerebrospinal fluid volume in people with restrained eating disorders compared to healthy controls, with a large effect size of 0.98, representing a very large increase (Figures 1, 2, 3).

#### Meta-analysis two: regional brain analysis of gray matter volume in females with AN

**Figure 4:**
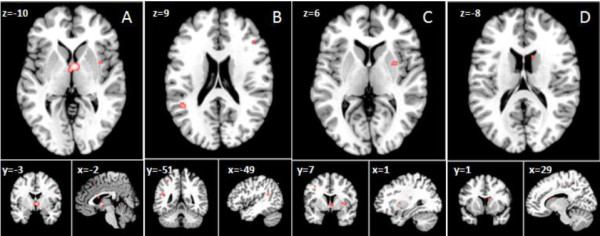
**Activation likelihood estimation of reduced regional brain volumes.** Showing clusters with significant ALE maxima (p < 0.05 for multiple comparisons, cluster size >100 mm^3^), Talairach coordinates are given for the respective slices. ALE clusters for the contrast AN < HC: (**A**) Hypothalamus; (**B**) L Inferior Parietal lobe. Brodmann area 39, (**C**) R Lentiform nucleus, (**D**) R Caudate.

From 47 foci, 222 subjects and 7 studies, demonstrating a regional decrease in gray matter volume, the ALE analysis revealed 4 significant clusters that survived the FDR threshold (Figure 4, Table 4). Cluster one was located in the Hypothalamus (x = −2, y = −3, z = −10), cluster two was in the left Inferior Parietal lobe (x = −49, y = −51, z = 9), cluster three, right lentiform nucleus (x = 1, y = 7, z = 6), cluster four was found in the right caudate (x = 29, y = 1, z = −8). For gray matter increase in people with AN we did not run a separate ALE meta-analysis because only one study reported the increase of certain region of gray matter in patient with AN compared to healthy controls [33].

#### Meta-analysis three: frequency data

**Figure 5:**
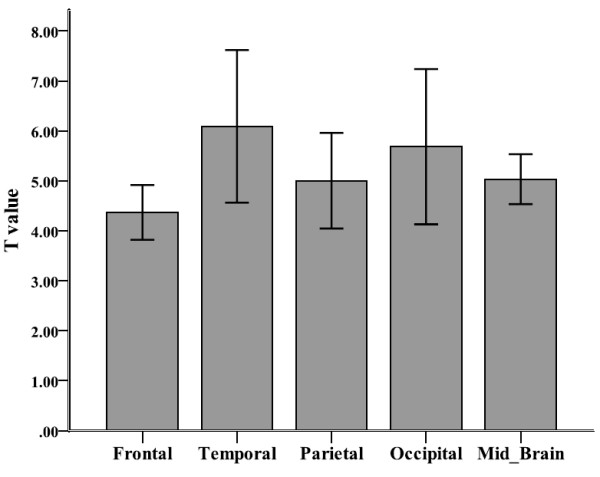
**Average T-Values across studies showing gray matter reduction in anorexia nervosa.** T value intensity of VBM studies showing regional gray matter decrease in people with anorexia nervosa and healthy controls.

**Figure 6:**
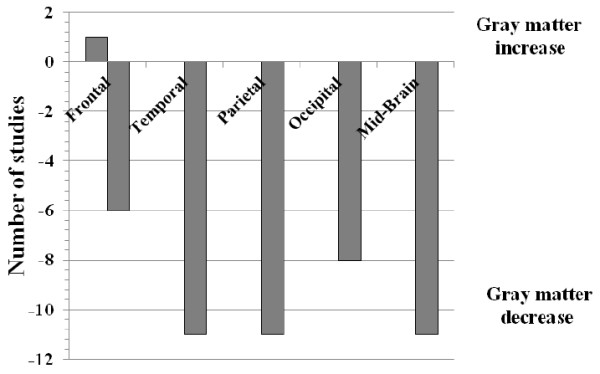
Number of studies demonstrating regional gray matter change in people with anorexia nervosa and healthy controls.

We used frequency data to examine both average T score intensity (to represent size of difference between case vs. control brain volume) and absolute number of studies reporting on specific brain regions, by aggregating T-statistics, generalising to a specific lobe of the brain (including mid-brain) and calculating an average score. We also did this for the number of studies (see Figures 5 and 6). We only used T-scores that were reported as significant in all papers. Below we report on these separate frequency analyses.

#### Average T scores

We found that the largest average T score values of gray matter decrease were found in temporal and occipital lobes in the group of people with AN. We present no average T score showing gray matter increase (only 1 study reported increase in the right DLPFC, Brooks et al., 2011).

#### Frequency of studies

Regional gray matter decrease in people with AN, was most frequently reported in the temporal lobe, parietal lobe, and mid-brain (study n = 11), as shown in Figure 6. One study reported increase in gray matter volume in people with AN.

#### Lateralization of brain changes

**Figure 7:**
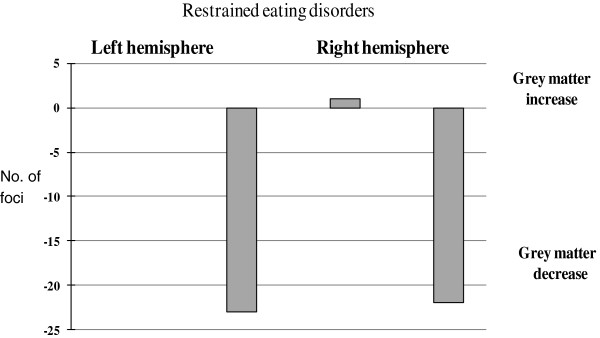
Regional grey matter change in people with anorexia nervosa in left and right hemispheres.

The analysis of regional gray matter change in left and right hemispheres showed that in those with AN gray matter is decreased almost equally in both hemispheres (study n = 22 versus n = 23) (Figure 7).

## Discussion

We demonstrate extensive global and regional meta-analyses comparing brain volume differences in patients currently ill with AN versus healthy controls in a total of 228 people with AN and 240 healthy controls. We show statistically significant data on global gray matter and white matter reductions; with concomitant increases in cerebrospinal fluid in those with AN. Broadly, the temporal and occipital lobes showed the most susceptibility to gray matter decrease in those currently ill with AN, and more studies report reduced volume in temporal and parietal cortices and mid-brain regions than others. Regionally, in those with AN, there were reductions in the hypothalamus, left inferior parietal lobe, right lentiform nucleus and right caudate. These regions are linked to appetite and somatosensory perception, functions which are often observed to be aberrant in those with AN. Hemispheric lateralisation did not seem to occur in those with AN, suggesting that starvation effects on the brain do not target one particular side of the brain.

The previous qualitative review [21] advised that future meta-analyses should include data on standard global analyses, taking into account BMI measures (e.g. comparing restrained vs. binge eaters), advice we heed here and extend by also conducting regional and frequency analyses. As was the case in the previous qualitative review, for our meta-analysis the sample heterogeneity prevented consideration of life-time history of eating disorders, psychiatric and medical comorbidity, handedness, eating disorder measures and medication use. Additionally, the ALE method does not enable covarying for other confounding factors, such as varying ages, sample sizes, different software packages used or other comorbidies, which is therefore a limitation of the ALE we present here. Perhaps future ALEs in the field, when more VBM experiments have been conducted, will be able to apply stricter selection of included studies. Our preliminary and novel meta-analyses concur with the previous qualitative review of VBM studies examining those with eating disorders, which cautiously concluded that their preliminary findings “…hint at gray matter reductions in people with anorexia nervosa, whereas the opposite (gray matter increase) may occur in people with binge eating [behaviour]”.

The authors of the previous review also conclude that “…efforts across the diagnostic eating disorder spectrum are likely to be most informative and such studies would allow for the investigation of the potential role of clinical and participant characteristics” [21]. This view is also in line with recent reviews discussing how updates to the DSM-IV will likely involve relaxing rigid diagnostic criteria in favour of a spectrum approach to classifying people with eating disorders [34,35]. Understanding which brain regions and their functions are more susceptible to over- or under eating might enable specific cognitive tests to gauge severity of eating pathology in order to rectify eating behaviour at an individual level. As part of this meta-analysis we also ran ALE on those with binge eating disorders, but given the heterogeneity of current VBM studies our data could not be included here (data available on request). However, it would be useful for future VBM studies to explicitly examine how varying levels of food consumption influence brain volume across the lifespan.

From the standpoint of under- versus normal/increased food consumption and the effects on brain volume, it is important to mention in the context of our meta-analyses that a recent VBM study comparing currently ill female adolescents with AN and following them through to recovery, showed results in a similar direction to ours [36]. Specifically, Mainz and colleagues showed GM reductions along the cortical midline that were reversible following recovery, with most significant increases in GM in the cerebellum, thalamus, hippocampus and amygdala, which also correlated with weight and hormone normalisation.

Considering volume differences in the context of functional MRI studies of those with AN may help to elucidate brain circuits most susceptible to acute malnutrition and the development of AN. In a recent review of neurobiological findings in AN [35] a summary of recent fMRI findings was given. Those with AN have reduced 3activation in *bottom-up* regions (e.g. mid-brain), such as the striatum, hippocampus, amygdala, hypothalamus and cerebellum, often in conjunction with increased *top-down* activation in prefrontal cortex regions such as the DLPFC, MPFC, ACC and OFC. Bottom-up activations are largely consistent with reward, motivation and general arousal, whereas top-down activations are linked to cognitive inhibition of appetite, self-referential goals and evaluation of salience. Additionally, the review highlights that brain imaging studies of AN also report aberrant activation in the insula, a temporal lobe structure associated with interoceptive awareness and cognitive/emotional perceptions of the body. Thus, our meta-analyses of reduced brain volume in bottom-up regions in those with AN are consistent with functional studies. During our review we found only one study demonstrating increased activation in the DLPFC in those with AN [33], suggesting that more VBM studies of AN need to closely examine PFC regions. Broadly, these structural and functional data in AN suggest that neural circuitry in the fronto-striatal pathway (linked to impulse control), which also involve connections with the insular cortex, is most susceptible to cognitively-maintained restraint of appetite in AN.

Our meta-analyses have comparable limitations to the recent qualitative review of VBM studies, such as differences in age, different analysis versions (although all studies used MRI), comorbidities with other disorders. Duration of illness, which was not taken into account may have been another factor in the differences observed in brain structure, one recent study of anorexia nervosa found no difference on brain structure between ‘short’ and ‘long’ duration of illness [4], whereas another recent VBM study found normalisation of brain regions on recovery [36]. An important strength of our work is that all the studies we included were corrected for total brain volume in the original publications. A further strength was that we conducted extensive meta-analyses from global, regional and frequency (T-scores and number of studies) perspectives. It would be beneficial for future VBM studies that examine the effects of food intake on the central nervous system to study otherwise healthy individuals (e.g. restrained eaters vs. overweight or food cravers), so that psychiatric and medical confounds can be avoided.

One seemingly conflicting finding in our analyses is that the ALE reports a significant reduction in the left inferior parietal lobe in patients with AN (Figure 4), whereas metric data of average difference scores (t-values) and number of reported studies per region (Figure 5. And Figure 6 respectively), does not. However, this merely highlights the significance of the ALE of reduced volume in the parietal lobe, which counts the number of foci reported across all studies, and weighs them up against other reported foci across the whole brain. It could be that there were fewer foci reported in other regions illustrated in the metric data, and also that the difference scores across all studies reporting reduced volume in the parietal lobe between AN patients and controls was consistently high. Given that the finding of reduced left inferior parietal lobe volume survived threshold-corrected whole brain ALE analysis, this should be taken as robust.

## Conclusions

The implications of our broad preliminary meta-analyses of structural brain differences in those with AN may have some clinical impact, given that we find specific regions that are seemingly more susceptible to a specific type of eating behaviour. In conjunction with the previous review, we find only gray matter that is reduced in those with AN compared to healthy controls, specifically in brain regions linked to appetite and somatosensory processing, functions which are consistently found to be aberrant in those with AN. It might be that prolonged restriction of food intake promotes abnormal reward responses to food and a deviation from a healthy feeling/perception of the body when eating. If there were more VBM studies examining those who have recovered from AN, which have shown so far normalisation across total brain volume, but remaining regional differences in the anterior cingulate, temporal lobe, parietal lobe and precuneus, e.g. [7], trait versus state effects might also be convincingly meta-analysed. Nevertheless, we present here for the first time a meta-analysis of structural brain differences that supports clinical observations of aberrant bodily perceptions and dysfunctional reward processing in AN, effects that are likely exacerbated by prolonged self-starvation.

## Competing interests

All authors declare no competing interests, financial or otherwise. All authors approve submission of the manuscript.

## Authors’ contributions

SJB devised the study and supervised OET, SJB, OCH and OET conducted the searches, analysed the data and illustrated the results, SJB, OET, OCH and HBS contributed to the writing of the manuscript. All authors read and approved the final manuscript.

## Pre-publication history

The pre-publication history for this paper can be accessed here:

http://www.biomedcentral.com/1471-244X/13/110/prepub

## Supplementary Material

Additional file 1: Table S1 Excluded studies from review and meta-analysis (n=13). Click here for file
