# Supplementary material for: Anorexia nervosa is linked to reduced brain structure in reward and somatosensory regions: a meta-analysis of VBM studies
Source: BMC Psychiatry. 2013 Apr 9;13:110. doi: 10.1186/1471-244X-13-110 (PMC3664070; doi:10.1186/1471-244X-13-110)
Supplement: Additional file 1: Table S1 — Excluded studies from review and meta-analysis (n=13). [file 1471-244X-13-110-S1.doc]

**Supplementary Table 1. Excluded studies from review and meta-analysis (n=13**)

| Study (author/year of publication Reason for exclusion | | |
| --- | --- | --- |
| Excluded from review (n=2) | |  |
| Van den Eynde et al., (2012) [1]  Frank et al., (2004) [2] | Review  Review |  |
| Excluded from Global analysis (n = 16) | |  |
| Wagner et al., (2006) [3]  Mühlau et al., (2007) [4]  **Joos et.al., (2010)** [5]*  **Brooks S. et al., 2011***  Suda et al., (2011) [6]  Frank G.K. (2007) | Recovered anorexic women  Recovered anorexic women  Global volumes are given as fractions  Not SPM approach  Only one patient with anorexia  Recovered anorexic women, no global brain volumes, not VBM |  |
| Excluded from ALE meta-analysis (n=15) |  |  |
| Wagner et al., (2006)  Mühlau et al., (2007)  **Roberto et al., (2011)** **  **Swayze et al., (2002)** **  Suda et al., (2011) [6]  Frank G.K. (2007) | No Talairach or MNI coordinates reported, recovered AN  Recovered anorexic women  No Talairach or MNI coordinates reported  No Talairach or MNI coordinates reported, not VBM  Only one patient with anorexia  Recovered anorexic women, no Talairach or MNI coordinates reported, not VBM |  |

* Publications, those were considered for ALE meta-analysis, but were excluded from Global analysis.

** Publications those were considered for Global analysis, but were excluded from ALE meta-analysis.

1. Van den Eynde F, Suda M, Broadbent H, Guillaume S, Van den Eynde M, Steiger H, Israel M, Berlim M, Giampietro V, Simmons A *et al*: **Structural magnetic resonance imaging in eating disorders: a systematic review of voxel-based morphometry studies**. *Eur Eat Disord Rev* 2012, **20**(2):94-105.

2. Frank GK, Bailer UF, Henry S, Wagner A, Kaye WH: **Neuroimaging studies in eating disorders**. *CNS Spectr* 2004, **9**(7):539-548.

3. Wagner A, Greer P, Bailer UF, Frank GK, Henry SE, Putnam K, Meltzer CC, Ziolko SK, Hoge J, McConaha C *et al*: **Normal brain tissue volumes after long-term recovery in anorexia and bulimia nervosa**. *Biol Psychiatry* 2006, **59**(3):291-293.

4. Muhlau M, Gaser C, Ilg R, Conrad B, Leibl C, Cebulla MH, Backmund H, Gerlinghoff M, Lommer P, Schnebel A *et al*: **Gray matter decrease of the anterior cingulate cortex in anorexia nervosa**. *Am J Psychiatry* 2007, **164**(12):1850-1857.

5. Joos A, Kloppel S, Hartmann A, Glauche V, Tuscher O, Perlov E, Saum B, Freyer T, Zeeck A, Tebartz van Elst L: **Voxel-based morphometry in eating disorders: correlation of psychopathology with grey matter volume**. *Psychiatry Res* 2010, **182**(2):146-151.

6. Suda M, Narita K, Takei Y, Aoyama Y, Takahashi K, Yuki N, Uehara T, Fukuda M, Mikuni M: **Changes in gray matter volume with rapid body weight changes in anorexia nervosa: a voxel-based morphometric study**. *Biol Psychiatry* 2011, **70**(8):e35-36.
